# Development and large-scale production of human milk fat analog by fermentation of microalgae

**DOI:** 10.3389/fnut.2024.1341527

**Published:** 2024-01-30

**Authors:** Xiaoying Zhou, Xinhua Zhao, Leon Parker, Paul Derkach, Mona Correa, Veronica Benites, Roberta Miller, Dino Athanasiadis, Bryce Doherty, Gawharah Alnozaili, Jon Wittenberg, Daniel Gates, Frédéric Destaillats, Walter Rakitsky, Scott Franklin

**Affiliations:** Checkerspot, Alameda, CA, United States

**Keywords:** human milk fat analog, infant nutrition, OPO, *sn*-2 palmitate, fermentation, structured TAG

## Abstract

**Background:**

Human milk contains a complex mixture of triacylglycerols (TAG), making it challenging to recreate using common ingredients.

**Objective:**

The study aimed to develop an innovative fermentation technique to produce essential human milk TAG, effectively tackling a significant hurdle in infant nutrition.

**Method:**

An in-depth analysis of the literature has been conducted to identify the specific TAG to be targeted. We used a microalgal oil production platform and a two-step procedure to modify its fatty acid and TAG composition. The palmitic acid (16:0) content has been increased by classical strain improvement techniques, followed by a step involving the expression of a lysophosphatidic acid acyltransferase (LPAAT) sequence capable of esterifying 16:0 specifically at the internal position (*sn*-2 palmitate) of TAG. Once the strain was stabilized, the fermentation was scaled up in a 50-L reactor to yield several kilograms of biomass. Subsequently, the oil was extracted and refined using standard oil processing conditions. Liquid chromatography-mass spectrometry was employed to monitor the TAG profile and the region specificity of 16:0 at the internal position (*sn*-2 palmitate) of TAG.

**Results:**

The initial strain had a 16:0 level of 25% of total fatty acids, which was increased to 30% by classical strain improvement. Simultaneously, the oleic acid level decreased from 61% to 57% of total fatty acids. Upon expression of an exogenous LPAAT gene, the level of the 16:0 esterified in the internal position of the TAG (*sn*-2 palmitate) increased by a factor of 10, to reach 73% of total palmitic acid. Consequently, the concentration of oleic acid in the internal position decreased from 81% to 22% of total fatty acids, with TAG analysis confirming that the primary TAG species in the oil was 1,3-dioleoyl-2-palmitoyl-glycerol (OPO). The 50-L-scale fermentation trial confirmed the strain's ability to produce oil with a yield of >150 g of oil per liter of fermentation broth in a timeframe of 5 days, rendering the process scalable for larger-scale industrialization.

**Conclusion:**

We have demonstrated the feasibility of producing a suitable TAG composition that can be effectively integrated into the formulations of infant nutrition in combination with other fats and oils to meet the infant feeding requirements.

## 1 Introduction

Human milk stands as an unparalleled, complex nutrient source, renowned for its intricate structural composition and diverse biological functionalities vital for infant development. Comprising 3%−5% of lipids, these nutrients serve as the primary caloric source for newborns, representing 50%−60% of the calories, or ~70 kcal/dl, in human milk (1). In breast milk, lipids are found as globules, predominantly composed of triacylglycerols (TAG), which represent ~98% of the total lipids (1). Human milk TAG contains a diverse range of fatty acids, spanning from caproic acid (6:0) to nervonic acid (24:1 n-9), with oleic, palmitic, and linoleic acids being the primary components (1). Notably, the TAG in human milk exhibits distinctive and conserved structures, characterized by the presence of palmitic acid at the internal position and unsaturated fatty acids like oleic and linoleic acids at the external positions (1–3). The specific configuration of palmitic acid in human milk TAG is also referred to as *sn*-2 palmitate, and this feature is largely unaffected by maternal diet variations worldwide and bestows several metabolic advantages, including enhanced absorption of TAG containing internal palmitic acid (4–6). This trait facilitates the absorption of essential nutrients such as calcium and liposoluble micronutrients while conferring immunological and anti-inflammatory properties in infants (5, 7, 8).

In situations where breastfeeding is not possible due to personal preferences and physiological or other constraints, providing safe alternatives with nutritional properties mirroring those of human milk becomes the alternative of choice for parents and caregivers. Over the years, numerous strategies have been explored to develop substitutes for human milk TAG. Enzymatic processes enabling the production of TAG rich in internal palmitic acid have been extensively employed in infant formula development (9). This method, categorized as a first-generation approach, has been in use for decades to improve the nutritional properties of infant formula (10–16). First-generation substitutes typically yield infant powders containing palmitic acid residing in the internal position at levels lower than 50% in commercial infant formula (9), which is a significant deviation from ~67.8% ± 4.7% found in human milk (17). These substantial variations in the repartition's level of palmitic acid concentration potentially hold crucial nutritional implications. Consequently, continuous research endeavors are essential to augment these developments, striving to achieve higher levels of palmitic acid at the internal level and thereby enhance the nutritional quality of infant products.

In response to this challenge, this study delves into the realm of fermentation, pioneering a groundbreaking methodology aimed at producing key TAG components of human milk, such as 1,3-dioleoyl-2-palmitoyl-glycerol (OPO). Through a meticulous review of the literature related to the complex fatty acid and TAG composition found in human milk, this research identifies a specific TAG as the target for replication. An innovative approach utilizing an oleaginous microalgae strain, which undergoes a series of modifications, including classical strain improvement techniques and the incorporation of specific genetic sequences, has been used. The resulting strain was used to produce a human milk fat analog on a large scale.

## 2 Materials and methods

### 2.1 Strain development

Modification of wild-type *Prototheca moriformis* strain isolate UTEX 1533 was performed to alter the stereoisomeric structure of TAG obtained by fermentation of *P. moriformis* to produce TAG enriched with palmitic acid (16:0) esterified to the middle position (*sn*-2) on the glycerol backbone. First, *P. moriformis* was subjected to classical mutagenesis to increase the level of palmitic acid from 25% to 30% of the total fatty acids. The classically improved strain was then genetically modified by targeted integration of a cassette encoding a heterologous lysophosphatidic acid acyl transferase (LPAAT), which is responsible for the regioselective insertion of saturated fatty acids (e.g., palmitic acid) at the *sn*-2 position of TAG produced by *P. moriformis*.

### 2.2 Production of algae oil

Algae oil was produced by fermenting the genetically modified strain of *P. moriformis* essentially as described in Running (18) in a 50-L-scale reactor, followed by the drying of algal biomass and subsequent mechanical and solvent extraction to recover crude oil. The latter was subsequently processed using standard edible oil refining methods, including degumming, bleaching, and deodorization.

### 2.3 Fatty acid and triacylglycerol analysis

Fatty acids are measured as their fatty acid methyl esters (FAMEs), following a direct transesterification reaction with a sulfuric acid methanol solution (19). The sample is injected on an Agilent 8890 gas chromatograph system equipped with a split/splitless inlet and flame ionization detector (Agilent Technologies, Palo Alto, CA, United States). An Agilent DB-WAX column (30 m × 0.32 mm × 0.25 μm dimensions) is used for the chromatographic separation of the FAME peaks. A FAME standard mixture purchased from Nu-Chek Prep (Nu-Chek Prep Inc., Elysian, MN, United States) is injected to establish retention times. Response factor corrections were previously determined empirically using various standard mixtures from Nu-Chek Prep. Methyl non-adecanoate (19:0) is used as an internal standard for the quantitation of individual FAMEs.

### 2.4 Determination of the *sn*-2 fatty acid profile

The fatty acid composition at the *sn*-2 position was determined after incubating the TAGs with porcine pancreatic lipase, as described by Christie and Han (19). The lipase reaction results in deacylation of the TAG at the *sn*-1(3) positions, leaving *sn*-2-MAGs. The *sn*-2-MAGs were then isolated using Agilent Bond Elut amino propyl solid phase extraction cartridges (Agilent Technologies, Palo Alto, CA, United States) and subjected to fatty acid composition analysis by gas chromatography as described earlier.

### 2.5 Triacylglycerol analysis

TAG profiles were determined as described previously using an Agilent 1290 Infinity II UHPLC system coupled to a 6470B triple quadrupole mass spectrometer and APCI ionization source according to the parameters described previously (23). Diacylglycerol (DAG) ion ratios, carried out as described by Byrdwell (24), were used to assess the relative abundance of TAG regio-isomers, and quantification was performed using a calibration curve of pure 1,3-dioleoyl-2-palmitoyl-glycerol (OPO), 1,2-dioleoyl-3-palmitoyl-glycerol (OOP), 1,2-dipalmitoyl-3-oleoyl-glycerol (PPO), and 1,3-dipalmitoyl-2-oleoyl-glycerol (POP) standards. For example, OPO (876.8 m/z) was monitored by the loss of oleic acid leading to the fragment at 577.5 m/z and the loss of palmitic acid leading to the fragment at 603.5 m/z.

## 3 Results

### 3.1 Identification of fatty acid and TAG compositional targets to guide the development of a new functional ingredient for infant nutrition

In human milk, lipids are primarily organized as globules known as milk fat globules (MFG), comprising a core of neutral TAG isolated by the layers of polar lipids, cholesterol, and transmembrane proteins (1, 25). TAG accounts for ~98% of MFG and consists of fatty acids with chain lengths ranging from 6 to 24 carbons (1, 25). The analysis of the variations of the fatty acid concentration measured as relative standard deviation percentage (RSD%; Table 1) in a dataset comprising more than 800 samples (20–22) reveals minimal variations (RSD <10%) in palmitic (16:0) and stearic (18:0) acids, supporting the hypothesis that these saturated fatty acids primarily originate from an endogenous pool generated through *de novo* synthesis. Oleic (18:1 n-9), γ-linolenic (18:3 n-6), and arachidonic (20:4 n-6) acids exhibit intermediate variation (<15%), indicating their relative insensitivity to ethnic and dietary differences. Conversely, medium-chain fatty acids like lauric (12:0) and myristic (14:0) acids, prevalent in certain tropical fats consumed significantly in the Philippines and Indonesia, have high coefficients of variation (>35%), as do linoleic (18:2 n-6) and γ-linolenic (18:3 n-3) acids, which are most abundant polyunsaturated fatty acids in vegetable oils, with coefficients of variation around 30%. Consistent with previous studies (26), concentrations of omega-3 long-chain polyunsaturated fatty acids, especially eicosapentaenoic (20:5 n-3) and docosahexaenoic (22:6 n-3) acids, vary considerably (coefficients of variation >50%) due to diverse marine product consumption patterns influenced by geographical origin and dietary habits of the studied populations (Table 1).

**Table 1:** Fatty acid composition of human milk samples (*N* = 835) collected in several countries^*^ (20–22). Results are expressed in g/100 g of fatty acids.

**Fatty acid**	**Mean**	**Median**	**Min**	**Max**	**SD**	**RSD%**
8:0	0.20	0.20	0.16	0.28	0.04	20.0
10:0	1.73	1.66	1.44	2.35	0.27	15.6
12:0	6.02	5.25	4.24	13.82	2.65	44.0
14:0	6.06	5.84	3.61	12.12	2.21	36.5
16:0	20.58	19.91	18.62	23.28	1.84	8.9
18:0	5.84	6.07	4.75	6.77	0.59	10.1
18:1 n-9	31.39	32.23	21.85	36.49	4.10	13.1
18:2 n-6	14.11	14.78	7.90	22.80	4.12	29.2
18:3 n-6	0.15	0.15	0.10	0.17	0.02	13.3
20:3 n-6	0.33	0.33	0.25	0.44	0.06	18.2
20:4 n-6	0.42	0.42	0.36	0.50	0.05	11.9
18:3 n-3	1.18	1.14	0.43	2.02	0.39	33.1
20:5 n-3	0.11	0.09	0.05	0.26	0.06	54.5
22:6 n-3	0.40	0.30	0.17	0.99	0.26	65.0
Other FA	10.08	10.13	-	-	-	-

Dataset consolidated from 835 samples of mature human milk collected in 10 counties as follows: Ref. 20: Australia (n = 48), Canada (n = 48), Chile (n = 50), China (n = 50), Japan (n = 51), Mexico (n = 46), Philippines (n = 54), the United Kingdom (n = 44), the United States (n = 49); Ref. 21: Singapore (n = 50); and Ref. 22: China (n = 345). SD stands for standard deviation, and RSD% stands for relative standard deviation percentage.

The examination of TAG molecular distribution, particularly the specific arrangement of fatty acids in internal (*sn*-2) or external [*sn-*1(3)] positions, revealed distinct asymmetry for palmitic, oleic, and linoleic acids, a characteristic observed not only in human milk but also in other mammals as reported decades ago (2, 3, 27). Advanced mass-spectrometry techniques have identified ~300 individual TAG species (Table 2), with the prominent ones being 1,3-dioleoyl-2-palmitoyl-glycerol (OPO) > 1-oleoyl-2-palmitoyl-3-linoleoyl-glycerol (OPL) > 1,2-dipalmitoyl-3-oleoyl-glycerol (PPO) > 1-oleoyl-2-palmitoyl-3-stearoyl-glycerol (OPS). Studies indicate regional differences, such as in China, where high vegetable oil consumption leads to elevated linoleic acid levels in human milk, resulting in higher concentrations of OPL compared to OPO (25, 28, 29). In all instances, palmitic acid is consistently esterified to the *sn*-2 position of TAG, constituting an average of 67.8 ± 4.7% of the total palmitic acid (Table 3), as determined through recent high-resolution mass spectrometry methods (17, 25, 28, 29).

**Table 2:** Main triacylglycerol (TAG) individual species determined by high-resolution mass-spectrometry techniques on 55 human milk samples (17).

**TAG**	**Average**	**SD**	**Norm. Average**
	**(% of total TAG)**		**(% of main TAG** ^*^ **)**
*sn*-OPO	13.9	2.1	41.2
*sn*-OPL	6.0	1.5	17.8
*sn*-PPO	5.7	1.6	16.9
*sn*-OPS	5.2	1.4	15.4
*sn*-POO	2.9	1.0	8.6

O, P, S, and L stand for oleic, palmitic, stearic, and linoleic acids, respectively. The prefix *sn*- indicates that the TAG species reported has a defined structure and is not a mixture of isomers.

^*^Normalized to the sum of *sn*-OPO, *sn*-OPL, *sn*-PPO, *sn*-OPS, and *sn*-POO as % of total TAG. Around 300 additional species have been reported in low numbers in this study. The normalized average has been estimated using the values for *sn*-OPO, *sn*-OPL, *sn*-OPP, *sn*-OPS, and *sn*-POO and used as indicative targets to guide the development of the new algae-derived human milk fat substitutes.

**Table 3:** The level of palmitic acid esterified in the internal (*sn*-2) and external [*sn*-1(3)] positions of the triacylglycerols (TAG) expressed as g/100 g of total palmitic acid determined by high-resolution mass-spectrometry techniques on 55 human milk samples (17).

**Palmitic acid**	**Average**	**SD**	**Min**	**Max**
*sn*-2 position	67.8	4.7	60.8	76.7
*sn*-1(3) position	32.2	4.7	23.3	39.2

Using the literature data presented in Tables 1–3, it is possible to establish compositional targets for the key fatty acids and the main TAG structures to guide the development of a new functional ingredient for infant nutrition. It is important to note that it is assumed that this ingredient's composition should emulate the structural complexity of TAG in breast milk when combined with other types of oils and fats to formulate a regulatory-compliant infant formula. Designing a strain capable of producing all the acids and TAG found in breast milk seems exceedingly challenging. Therefore, the concentration of the palmitic acid set is higher compared to human milk data to account for the effect of the dilution with other vegetable oils that might be used in the final oil mix. We assumed that the new ingredient might represent 40–60% of the total lipid composition of infant formula and used the following targets for strain development:

- OPO: 40 ± 3% of total TAG in accordance with literature data (Table 2).

- Palmitic acid: 30 ± 3% of total fatty acids, of which 67.8 ± 4.7% in the internal position of TAG in accordance with literature data (Table 3).

Due to significant fluctuations in the levels of polyunsaturated fatty acids, which are regulated differently in infant formula across countries, there are no fixed targets in place. We assumed that easily accessible sources like canola, soybean, DHA, and ARA microbial oils could be utilized to create a nutritionally complete and regulation-compliant oil mixture.

### 3.2 Development of the strain and production of the algae oil

A wild-type *P. moriformis* isolate (UTEX 1533) was mutagenized utilizing classical strain improvement to increase levels of palmitate beyond the 25% normally seen in this strain. The resulting mutant strain (named the high-palmitic acid strain) was capable of reproducibly elaborating over 30% palmitate, as shown in Table 4. However, regiospecific lipase treatment, followed by FAME analysis of the resulting monoacylglycerols, showed only modest changes in fatty acid distribution along the glycerol backbone. This was in contrast to the oil produced by the strain subsequent to the introduction of the heterologous LPAAT. While palmitate levels remained relatively constant between the classically improved isolate and the transgenic strain, regiospecific insertion of saturates shifted dramatically, with *sn-2* levels of palmitate increasing from 6% to 69.3% in the transgenic line. Not surprisingly, *sn*1–3 insertion of palmitate decreased over 10-fold in the transgenic line, dropping from 35% to 3.3%. The 50-L-scale fermentation trial conclusively demonstrated the strain's capacity for oil production, achieving a yield of over 150 g of oil per liter of fermentation broth within 5 days. This outcome underscores the strain's efficiency and establishes the feasibility of upscaling the process.

**Table 4:** Fatty acid profiles of total triacylglycerols (TAG) as well as *sn*-1(3) and *sn*-2 positions from the parent *P. moriformis* microalgae strain, the high palmitic acid strain generated by mutagenesis, and the OPO strain engineered by insertion of the LPAAT gene.

	**Parent strain**			**High-palmitic acid strain**			**OPO-engineered strain**		
**Fatty acid**	**TAG**	***sn*** **-1(3)**	***sn*** **-2**	**TAG**	***sn*** **-1(3)**	***sn*** **-2**	**TAG**	***sn*** **-1(3)**	***sn*** **-2**
16:0	25.3	36.5	3	30.3	35	6	31.5	3.3	69.3^*^
18:0	3.3	4.7	0.5	2.3	4.7	0.5	2.6	4.7	0.5
18:1 n-9	61.1	48.9	85.6	57.4	50.8	81.6	55.2	80.3	22.8
18:2 n-6	6.9	6.1	8.4	6.3	6	8.5	6.3	8.4	3.7
Other FA	3.4	3.9	2.5	3.8	3.5	3.3	4.4	3.3	3.7

^*^When expressed in g/100 g of total palmitic acid, the level of palmitic acid esterified in the internal (sn-2) position is 73.3%. Data are expressed in g/100 g of total fatty acids.

Table 5 illustrates the TAG profile of the resulting engineered strain vs. oils found in several commercially available OPO ingredients. *sn*-2 analysis shows that oil from the engineered microalgal strain has higher *sn*-2 palmitate levels than any of the commercial infant fat formulations tested (73.3% vs. 38.95% in the commercial fat containing the lowest level of palmitate at the *sn*-2 position). Similarly, regiospecific OPO levels in oil derived from the transgenic strain were higher than any of the commercial oils, at 38.3% vs. 21.7% in the commercial fat containing the lowest level of OPO. While levels of highly desirable OPO were higher in oil derived from the transgenic line, levels of less desirable TAG species, including OOP, PPO, and PPP, were lower in algae oil compared to any of the commercial OPO ingredient samples tested (Table 5).

**Table 5:** Total *sn*-2 palmitate and main triacylglycerols (TAG) of individual species of the algae-derived human milk fat substitute developed in the present study (OPO-engineered strain) and in commercial human milk fat substitute samples (CS1–3). TAG results are expressed in g/100 g of TAG and *sn*-2 and *sn*-1(3) palmitate data in g/100 g of total palmitic acid.

**TAG**	**OPO-engineered strain**	**CS1**	**CS2**	**CS3**
*sn*-2 palmitate	73.30	65.44	55.93	38.95
*sn*-1(3) palmitate	26.70	34.56	44.07	61.05
*sn*-OPO	38.30	34.40	30.20	21.70
*sn*-OOP	2.40	3.30	4.70	5.90
*sn*-PPO	10.90	14.60	13.30	20.30
*sn*-POP	0.40	0.00	1.00	3.00
OOO	13.17	9.67	11.10	11.02
OPL	9.56	13.21	12.89	8.14
POS	3.91	5.88	4.81	6.44
OOM	2.66	0.00	0.00	0.42
OOL	4.88	4.81	6.23	3.28
MOP	2.14	0.00	0.00	0.00
LOL	1.31	0.92	1.47	0.69
MOL + POLn	1.22	0.00	0.00	0.00
OOS + POG	1.52	2.83	2.88	2.85
PPL	1.22	2.42	2.80	2.99
PPP	0.00	1.57	1.27	5.30
Other species	4.30	4.32	4.18	5.65

O, P, S, L, M, Ln, and G stand for oleic, palmitic, stearic, linoleic, myristic, a-linolenic, and gondoic acids, respectively. The prefix *sn*- indicates that the TAG species reported has a defined structure and is not a mixture of isomers.

## 4 Discussion

The design of infant formula presents a challenge in mimicking the composition and functional properties of human milk TAG. Understanding the structure-function relationships of human milk TAG is essential for formulating nutritionally optimized infant formulas that closely mimic the benefits of human milk (25). Various strategies have been explored to incorporate human milk-mimicking TAG structures into infant formulas, including lipid blending, interesterification, and structured lipid synthesis (25).

**Figure 1:**
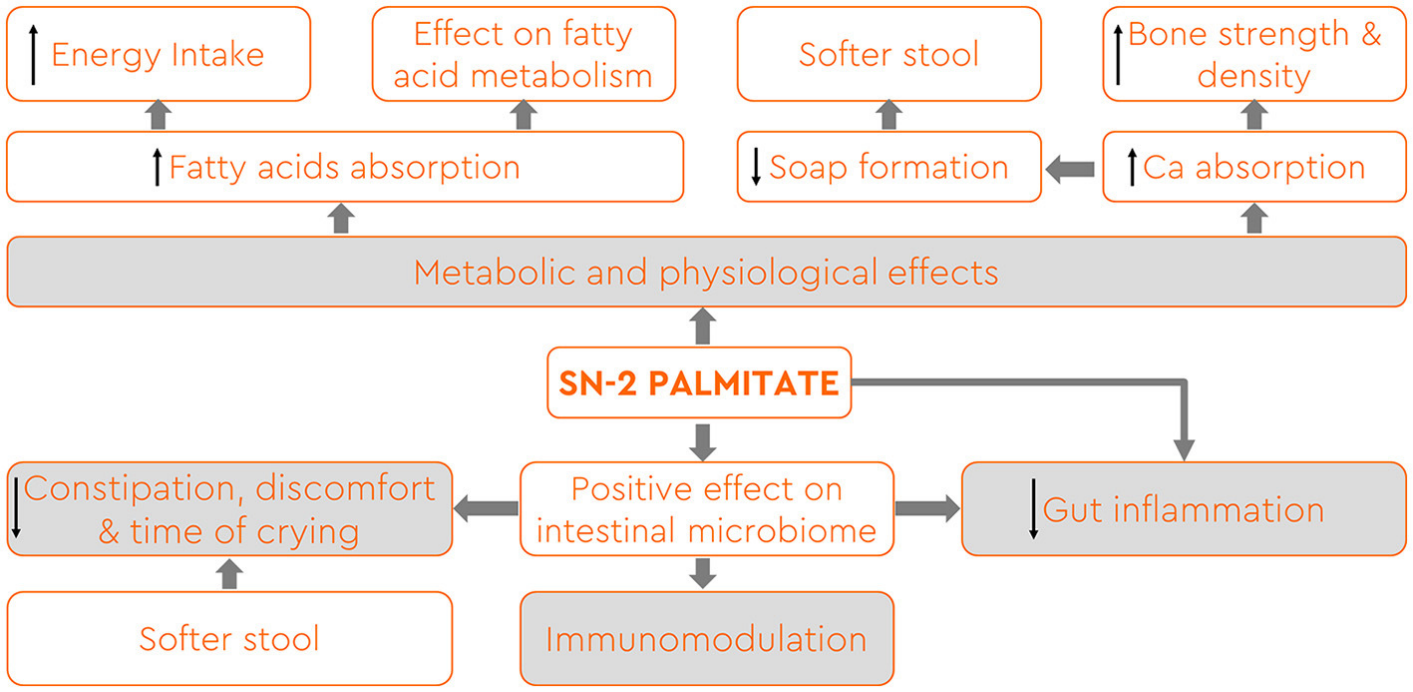
Health benefits associated with the occurrence of palmitic acid esterified in the internal position of TAG (*sn*-2 palmitate) in breast milk and infant formula (adapted from references 5 and 8).

In recent years, considerable research efforts have focused on unraveling the complexities of human milk TAG and elucidating their impact on infant nutrition and health (25). Havlicekova et al. (5) as well as Miles and Calder (8) summarized in a very comprehensive review all the health benefits that emerged from investigating in animals and premature as well as term-born infants the effects of higher *sn*-2 palmitate content in supplemental milk formula (Figure 1). The positional distribution of fatty acids within TAG is known to influence their digestion, absorption, and subsequent utilization by infants. The presence of palmitic acid at the *sn*-2 position (*sn*-2 palmitate) enhances the formation of stable emulsions during digestion, facilitating efficient lipid absorption. *sn*-2 palmitate improved fatty acid absorption and reduced the formation of insoluble calcium dipalmitate, leading to softer stools without increased volume (4, 12–15, 30–35). Studies also revealed a direct relationship between *sn*-2 palmitate content and calcium absorption, reducing calcium waste in stools and improving overall mineral balance (7, 36–38). Infants fed high *sn*-2 palmitate formula displayed significantly higher bone mineral mass compared to those fed standard formula, akin to breast-fed infants (13, 39, 40). While studies indicated a positive impact on bone health, further research is needed to assess long-term effects. Some studies suggest that infants fed formula enriched with *sn-*2 palmitate experienced reduced crying episodes and duration, especially during evenings and nights (10, 41–43). This effect may be partially attributed to a synergetic interplay between *sn*-2 palmitate, prebiotic oligosaccharides, and whey hydrolysate that have been used in combinations in these studies (41). *sn*-2 palmitate influenced the intestinal microbiome positively, increasing beneficial bacteria such as *Lactobacillus* and *Bifidobacteria in vitro* (25, 44–47) and *in vivo* settings (16, 33, 48, 49). This modulation of the microbiome had immunomodulatory effects and positively influenced intestinal maturation, counteracting the growth of pathogenic bacteria (44, 50–53). Experimental studies demonstrated that *sn*-2 palmitate might have a protective effect against intestinal inflammation (25, 54). Animal models showed reduced intestinal erosion and other morphological changes in mice fed a high *sn*-2 palmitate formula, suggesting potential anti-inflammatory properties (54). Further research is necessary to fully explore the long-term impact of this aspect.

All these studies were conducted using human milk fat substitutes produced enzymatically using vegetable oils, particularly palm oil fractions, as sources of oleic and palmitic acids (9). This method was historically developed by Bungee Loders Crocklaan and Enzymotec and has been scaled up to produce various formulations under commercial names such as Betapol™ and Infat™. With this approach, it is possible to obtain oils with palmitic acid content in the *sn*-2 position ranging from 39% to 65% of the total palmitic acid content [(25), Table 5]. These concentrated oils are subsequently used as blends in combination with other oils such as coconut oil, high-oleic sunflower oil, and canola oil to achieve a nutritionally balanced composition in compliance with relevant regulations. The emergence of these *sn*-2 palmitate concentrates has enabled a deeper understanding of their nutritional effects and has contributed to advancements in our comprehension of the relationship between the complex TAG structures in human breast milk and their nutritional functions. Nonetheless, significant differences exist in nutritionally relevant clinical endpoints, such as fatty acid absorption, residual calcium in feces, and stool consistency, when comparing infant formulas containing these first-generation and breast-fed infants (4, 12–15, 31, 33, 36). Human milk fat substitutes produced by enzymatic methods contain significant levels of TAG, such as 1,3-dipalmitoyl-2-oleoyl-glycerol (POP), 1,2-dipalmitoyl-3-oleoyl-glycerol (PPO), 1,2-dipalmitoyl-2-linoleoyl-glycerol (PPL), 1-palmitoyl-2-oleoyl-3-stearoyl-glycerol (POS), and tripalmitin (PPP, Table 5), which are not present in such levels in breast milk (Table 2). The digestion of these TAGs, upon hydrolysis by various lipases active in newborns, results in the release of palmitic acid in the digestive tract (25). These free fatty acids can combine with divalent minerals, such as calcium, to form insoluble calcium dipalmitate that is excreted in feces (25).

To address the challenge of residual palmitic acid accumulation in the external position of TAG and the desire to achieve a high *sn*-2 palmitate content, it is crucial to minimize the use of palmitic-rich fractions derived from tropical fats for enzymatic human milk fat substitute synthesis. Instead, we explore an innovative approach by leveraging the potential of oleaginous microalgae. Although microalgae present a promising avenue for the controlled production of TAG oils, a significant challenge in developing a viable substitute for human milk fat lies in the inherent composition of TAG produced by microalgae as well as the low lipid productivity of these microorganisms (55). Unlike human breast milk and certain animal fats, microalgae and higher plants typically synthesize TAG with unsaturated fatty acids in the internal position and saturated fatty acids in the external position. To address this disparity, our research initially focused on enhancing the palmitate content, which was originally 25.3% in the parent strain (Table 4). Utilizing classical strain improvement techniques, we successfully increased it to slightly over 30% (Table 4). Subsequently, genetic transformations were employed to modify the esterification selectivity of saturated fatty acids on the glycerol backbone, favoring the production of TAG where palmitic acid predominantly occupied the internal position. This strategic approach led to the isolation of a strain consistently producing oil with a high palmitic acid concentration, reaching 69.3% (Table 4), esterified to the internal position. When normalizing the palmitic acid concentration in the *sn*-2 position to the total concentration of palmitic acid in the TAG, we achieved a level of 73.3% (Table 5). Notably, the primary TAG in this oil was identified as OPO, constituting ~38.3% of the total TAG produced, as illustrated in Table 5.

**Figure 2:**
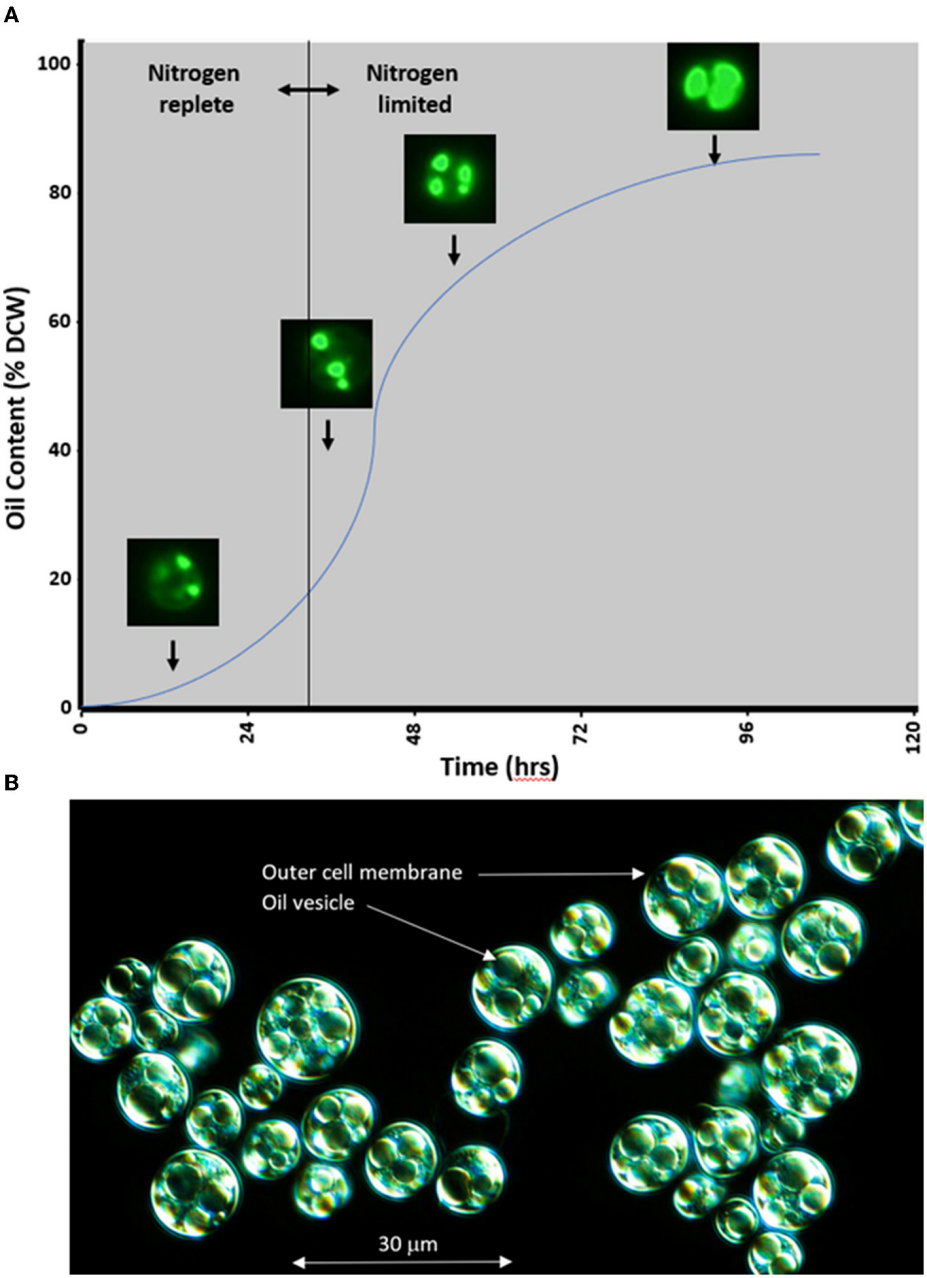
**(A)** Schematic representation of oil production kinetics in heterotrophic microalgae *Prototheca moriformis*. Microalgal cells stained with BODIPY^TM^ 493/503 (4,4-Difluoro-1,3,5,7,8-Pentamethyl-4-Bora-3a,4a-Diaza-s-Indacene) and imaged under fluorescence show increasing oil accumulation in response to nitrogen deprivation in a time-dependent manner. This process is reversible, and if nitrogen is returned to the system, algae will catabolize their lipid stores and re-enter a state of vegetative reproduction. **(B)** Micrograph of microalgae *P. moriformis* at the end of fermentation, displaying intracellular large oil vesicles.

Microalgae species like *P. moriformi*s, used in the present study, exhibit a doubling time compared to that of yeast (around 3–4 h) when cultivated in a defined medium containing suitable sugars and the same macro- and micronutrients used by terrestrial plants. When nitrogen becomes scarce in the presence of sugars, oleaginous microalgae halt their division and transition their metabolism toward oil production, as illustrated in Figure 2A. This process involves the conversion of carbon from sugars into fatty acids within the plastid, followed by their incorporation into TAG within the endoplasmic reticulum. These TAG accumulate within the cell in the form of large vesicles, leading to biomass, where ~80% of the dry cell weight consists of oil, as depicted in Figure 2B. Furthermore, this oil contains various micronutrients such as tocopherols, tocotrienols, and sterols, the composition of which varies based on the strain. Industrial-level oil production from microalgae involves specific fermentation conditions spanning 3–5 days (Figure 2A). After this period, the fermentation broth is harvested and dried to obtain biomass, from which oil can be extracted using conventional pressing or solvent extraction methods. The resulting crude oil is then refined using standard techniques to yield a neutral-tasting and neutral-colored oil suitable for the formulation of nutritional products. It is imperative to emphasize that microalgae oils are cultivated under controlled fermentation conditions, allowing for a highly traceable supply chain designed to minimize exposure to chemical, microbial, and allergenic contaminants that could pose adverse health effects. A comprehensive food safety evaluation will be essential before introducing this new ingredient to the market, ensuring compliance with regulatory approvals in various legislations.

## 5 Conclusion

The structural variations observed in TAG within human milk hold profound implications for infant health and development, particularly impacting processes like digestion, absorption, modulation of gut microbiota, immune function, and potentially long-term health outcomes. To emulate these structure-function relationships in human milk TAG, infant formulas aim to deliver optimal nutrition and promote the overall wellbeing of infants who are not exclusively breastfed. The utilization of fermentation technology signifies a significant stride in the pursuit of creating nutritionally relevant and structurally analogous analogs for human milk fat. We demonstrated that this technology enables the precise targeting of specific TAG structures, such as OPO, while minimizing the presence of TAG species like PPO, POP, POS, and PPP, which produce calcium dipalmitate, an anti-nutritional compound that hinders the optimal utilization of fatty acids and minerals in infants.

## Data availability statement

The analytical raw data supporting the conclusions of this article will be made available by the authors, without undue reservation.

## Author contributions

XZho: Writing—original draft, Formal analysis, Investigation, Methodology, Resources, Supervision. XZha: Formal analysis, Investigation, Methodology, Resources, Writing—original draft. LP: Formal analysis, Investigation, Methodology, Resources, Project administration, Supervision, Validation, Writing—original draft. PD: Formal analysis, Resources, Writing—original draft. MC: Formal analysis, Resources, Methodology, Writing—original draft. VB: Methodology, Resources, Writing—original draft. RM: Resources, Writing—original draft. DA: Formal analysis, Methodology, Resources, Writing—original draft. BD: Resources, Writing—original draft. GA: Writing—original draft, Resources. JW: Resources, Writing—original draft. DG: Writing—review & editing, Formal analysis, Methodology, Resources. FD: Conceptualization, Writing—review & editing, Writing—original draft. WR: Conceptualization, Writing—review & editing. SF: Conceptualization, Investigation, Methodology, Project administration, Writing—review & editing.
